# Racial and Ethnic Disparities in Alcohol-Attributed Deaths in the United States, 1999–2020

**DOI:** 10.3390/ijerph20085587

**Published:** 2023-04-20

**Authors:** Ibraheem M. Karaye, Nasim Maleki, Ismaeel Yunusa

**Affiliations:** 1 Department of Population Health, Hofstra University, Hempstead, NY 11549, USA; 2 Department of Psychiatry, Harvard Medical School, Massachusetts General Hospital, Boston, MA 02114, USA; 3 Department of Clinical Pharmacy and Outcomes Sciences, University of South Carolina, Columbia, SC 29208, USA

**Keywords:** alcohol, disparities, mortality, trends, burden, United States, differences, epidemiology

## Abstract

The disparities in alcohol-attributed death rates among different racial and ethnic groups in the United States (US) have received limited research attention. Our study aimed to examine the burden and trends in alcohol-attributed mortality rates in the US by race and ethnicity from 1999 to 2020. We used national mortality data from the Centers for Disease Control and Prevention’s Wide-ranging Online Data for Epidemiologic Research (CDC WONDER) database and employed the ICD-10 coding system to identify alcohol-related deaths. Disparity rate ratios were calculated using the Taylor series, and Joinpoint regression was used to analyze temporal trends and calculate annual and average annual percentage changes (APCs and AAPCs, respectively) in mortality rates. Between 1999 and 2020, 605,948 individuals died from alcohol-related causes in the US. The highest age-adjusted mortality rate (AAMR) was observed among American Indian/Alaska Natives, who were 3.6 times more likely to die from alcohol-related causes than Non-Hispanic Whites (95% CI: 3.57, 3.67). An examination of trends revealed that recent rates have leveled among American Indians/Alaska Natives (APC = 17.9; 95% CI: −0.3, 39.3) while increasing among Non-Hispanic Whites (APC = 14.3; 95% CI: 9.1, 19.9), Non-Hispanic Blacks (APC = 17.0; 95% CI: 7.3, 27.5), Asians/Pacific Islanders (APC = 9.5; 95% CI: 3.6, 15.6), and Hispanics (APC = 12.6; 95% CI: 1.3, 25.1). However, when the data were disaggregated by age, sex, census region, and cause, varying trends were observed. This study underscores the disparities in alcohol-related deaths among different racial and ethnic groups in the US, with American Indian/Alaska Natives experiencing the highest burden. Although the rates have plateaued among this group, they have been increasing among all other subgroups. To address these disparities and promote equitable alcohol-related health outcomes for all populations, further research is necessary to gain a better understanding of the underlying factors and develop culturally sensitive interventions.

## 1. Introduction

Examining the differences in alcohol consumption, alcohol-related disorders, and health consequences across racial and ethnic groups is an important and continually evolving field of research [1,2,3]. Despite widespread awareness of alcohol’s harmful effects on health, it has become apparent that these effects are not equally distributed among racial and ethnic groups in the United States (US), much like other health outcomes [3,4,5,6,7,8].

The current literature indicates that Native Americans and Hispanics tend to have the highest rates of alcohol consumption, with variations within these groups (e.g., based on age and gender) [3,9]. On the other hand, higher rates of alcohol use disorders have been reported among Whites and Native Americans [3,10]. However, in terms of alcohol dependence, Blacks and Hispanics experience higher rates of relapse [3,11,12,13,14]. Moreover, health issues related to alcohol use appear to be more prevalent among Native Americans, Hispanics, and Blacks [3,15]. Disparities in accessing care and treatment for alcohol-related problems are especially pronounced among Hispanics [3]. The causes of these disparities are complex and remain unclear, potentially reflecting differences in drinking behaviors, alcohol accessibility, exposure to racial/ethnic discrimination, access to care, cultural factors, economic and neighborhood disadvantages, and variations in alcohol-metabolizing genes [3,16,17].

Despite the nuanced, complex, and uncertain understanding of racial and ethnic disparities in alcohol-related use and consequences, there is a notable deficiency of published studies assessing these disparities, particularly on alcohol-related deaths. Previous studies have primarily focused on disparities in alcohol consumption, treatment, and use disorders [17,18,19,20], and the limited available studies that have analyzed disparities in alcohol mortality either date back to several years ago [21] or have only analyzed data from specific states, instead of providing a national perspective [22]. To comprehensively understand these disparities and progress toward health equity, it is necessary to investigate how they manifest across a range of health outcomes, including mortality. This study utilizes national mortality data from the Centers for Disease Control and Prevention (CDC) to examine disparities in the burden and trends of alcohol-related mortality among racial and ethnic groups in the US from 1999 to 2020. The findings could help inform public health policies and interventions that are culturally sensitive and promote equitable alcohol-related health outcomes for all populations. By shedding light on these disparities, this study could also contribute to the larger conversation on health equity and the need for equal access to care and services for vulnerable populations.

## 2. Materials and Methods

### 2.1. Data Sources

This study utilized national mortality data from the CDC’s Wide-ranging Online Data for Epidemiologic Research (WONDER) database (1999–2020) [23]. The underlying cause of death was identified as related to alcohol using the International Classification of Diseases codes, 10th Revision—E24.4 (alcohol-induced pseudo-Cushing syndrome), F10 (alcohol-related disorders [that includes mental and behavioral disorders due to alcohol use]), G31.2 (degeneration of the nervous system due to alcohol), G62.1 (alcoholic polyneuropathy), G72.1 (alcoholic myopathy), I42.6 (alcoholic cardiomyopathy), K29.2 (alcoholic gastritis), K70 (alcoholic liver disease), K85.2 (alcohol-induced acute pancreatitis), K86.0 (alcohol-induced chronic pancreatitis), R78.0 (finding of alcohol in the blood), X45 (accidental poisoning by and exposure to alcohol), X65 (intentional self-poisoning by and exposure to alcohol), and Y15 (poisoning by and exposure to alcohol, undetermined intent) [23]. The age-adjusted mortality rate (AAMR) was extracted based on demographic factors, including race/ethnicity (Non-Hispanic Whites; Non-Hispanic Blacks; Hispanics; Asians/Pacific Islanders; American Indians/Alaska Natives), age (15–24; 25–44; 45–64; ≥65), sex (male; female), census region (Northeast; Midwest; South; West), and cause of injury (alcohol poisoning; other causes). Data extraction was supervised by the interdisciplinary authors’ team to ensure accuracy and consistency in the process. We used the direct method to calculate the AAMR, which involves applying age-specific death rates from the study population to the standard population. This method allowed the study to account for differences in the age distribution of each racial and ethnic group, which is crucial when comparing mortality rates across populations with different age structures.

WONDER is a publicly accessible, de-identified database, so this study was exempted from institutional review board review.

### 2.2. Statistical Analysis

The disparity rate ratio was calculated by dividing the AAMR of each racial/ethnic subgroup by the AAMR of the White population. The 95% confidence interval of the rate ratio was determined using Taylor’s series expansion. To examine the temporal trends, a Joinpoint regression model was fitted. The model initially assumes that the AAMR is linear, i.e., without any Joinpoint, and that the rate of change is consistent throughout the study period [24]. A Joinpoint is then included to indicate an alteration in trend, and a statistical method is used to test the significance of the model relative to the initial null model. If the inclusion of the Joinpoint improves the model, it is retained; otherwise, it is excluded. This process was repeated through 4499 Monte Carlo permutations until the final model was derived. Finally, the 95% confidence intervals were calculated using the parametric method.

Additional detail regarding Joinpoint analysis is available elsewhere [25].

### 2.3. Model Specifications/Parameter Settings

The log-transformed AAMR was modeled as the outcome variable, and the year of death was used as the independent variable. The interval type was set as annual to evaluate the yearly change in AAMR during the study period. The errors option was set as constant variance (homoscedastic) to align with the constant variance of the standard errors, as assessed using the Breusch Pagan test. In addition, default options were selected for the method (grid search), number of Joinpoints (range 0 to 4), model selection method (permutation test), Average Annual Percentage Changes (AAPC) segment ranges (1999 to 2020), Annual Percentage Changes (APC)/AAPC/Tau confidence intervals (parametric method), permutation test (4499), and overall significance level (0.05).

All statistical analyses were performed using Stata 17, the Joinpoint Regression Program (version 4.9.1.0), and the Open-Source Epidemiologic Statistics for Public Health software (version 3.01).

## 3. Results

Between 1999 and 2020, a total of 605,948 individuals died from alcohol-related causes in the US. The highest AAMR was observed among American Indian/Alaska Natives, who were 3.6 times as likely (95% CI: 3.57, 3.67) to die from alcohol-related causes compared to Non-Hispanic Whites. Non-Hispanic Blacks, Asians/Pacific Islanders, and Hispanics showed lower AAMRs compared to Non-Hispanic Whites. These results were similar when stratified by sex, with American Indian/Alaska Native males being 3.2 times as likely (95% CI: 3.09, 3.21) and females being 4.8 times as likely (95% CI: 4.73, 4.96) to die from alcohol compared to Non-Hispanic Whites.

Among individuals aged 15–24 years, American Indians/Alaska Natives were 12.2 times as likely (95% CI: 10.80, 13.67) to die from alcohol compared to Non-Hispanic Whites, and Hispanics were 1.7 times as likely (95% CI: 1.56, 1.84). Among those aged 45–64 years, American Indians/Alaska Natives (AAMR = 84.5; 95% CI: 82.9, 86.1) and Hispanics (AAMR = 23.8; 95% CI: 23.5, 24.0) had the highest AAMRs. The highest AAMR among individuals aged 25–44 years was among American Indians/Alaska Natives (AAMR = 47.4; 95% CI: 46.2, 48.5), who were 7.8 times as likely to die (95% CI: 7.61, 7.99) than Non-Hispanic Whites. Older adults showed the highest AAMR among American Indians/Alaska Natives (AAMR = 41.6; 95% CI: 39.8, 43.4), followed by Hispanics (AAMR = 20.2; 95% CI: 19.9, 20.6) and Non-Hispanic Blacks (AAMR = 13.5; 95% CI: 13.3, 13.8), with the lowest AAMR observed among Asians/Pacific Islanders (AAMR = 3.7; 95% CI: 3.5, 3.9).

When stratified by census region, American Indians/Alaska Natives recorded the largest AAMR across all regions, being 1.1 times as likely to die (95% CI: 1.02, 1.28) than Non-Hispanic Whites in the Northeast, 4.1 times as likely in the Midwest (95% CI: 4.00, 4.27), 1.4 times as likely in the South (95% CI: 1.36, 1.48), and 3.7 times as likely in the West (95% CI: 3.67, 3.80). Results were similar when stratified by cause of death, with the highest AAMR among American Indians/Alaska Natives being from alcohol poisonings (AAMR = 4.2; 95% CI: 4.0, 4.3) and all other causes (AAMR = 34.5; 95% CI: 34.0, 35.0) (Table 1).

### Temporal Trends

From 1999 to 2020, overall mortality trends remained stable until 2007 (APC = 0.0; 95% CI: −0.6, −0.6), and then increased by 3% per year (95% CI: 2.6, 3.5) from 2007 to 2018 and by 14% per year (95% CI: 8.2, 20.3) from 2018 to 2020. Among all racial/ethnic subgroups, trends increased in recent years, except among American Indians/Alaska Natives, whose rates remained stable from 2018 to 2020 (APC = 17.9; 95% CI: −0.3, 39.3). Specifically, the most recent trends in alcohol-related mortality, from 2018 to 2020, show an annual increase of 14.3% among Non-Hispanic Whites (95% CI: 9.1, 19.9), 12.6% among Hispanics (95% CI: 1.3, 25.1), and 17% among Non-Hispanic Blacks (95% CI: 7.3, 27.5). For Asians/Pacific Islanders, the trend has worsened by 9.5% per year between 2015 and 2020 (95% CI: 3.6, 15.6).

When stratified by sex, recent mortality trends increased among all racial/ethnic subgroups. For males, the trends increased at a rate of 13.3% among Non-Hispanic Whites from 2018 to 2020 (95% CI: 7.3, 19.5), 15.8% among Non-Hispanic Blacks from 2018 to 2020 (95% CI: 3.3, 29.7), 3.1% among Hispanics from 2012 to 2020 (95% CI: 1.6, 4.7), 10.4% among Asians/Pacific Islanders from 2016 to 2020 (95% CI: 2.0, 19.5), and 4.2% among American Indians/Alaska Natives from 2006 to 2020 (95% CI: 3.2, 5.3). For females, recent trends increased by 15% per year (95% CI: 8.1, 22.4) among Non-Hispanic Whites, 17% among Non-Hispanic Blacks (95% CI: 0.3, 36.4), 5.5% among Asians/Pacific Islanders (95% CI: 3.8, 7.2), 22.8% among American Indians/Alaska Natives (95% CI: 5.9, 42.4), and 2.9% among Hispanics (95% CI: 2.0, 3.8).

When stratified by age, recent trends among Non-Hispanic Whites aged 15–24 remained stable from 2018 to 2020 (APC = 26.0; 95% CI: -8.6, 73.6), while trends increased among Hispanics of the same age group by 5.2% from 2013 to 2020 (95% CI: 1.1, 9.5). Among individuals aged 25 and above, recent trends increased across all racial/ethnic subgroups. For instance, among individuals aged 25 to 44 years, the most recent trend, from 2018 to 2020, has shown an annual increase of 24.4% among Non-Hispanic Whites (95% CI: 14.0, 35.7), 29.4% among Non-Hispanic Blacks (95% CI: 7.0, 56.5), 24.3% among American Indians/Alaska Natives (95% CI: 6.9, 44.6), and 25.4% among Hispanics (95% CI: 9.3, 43.8). Similarly, the recent trend for Asians/Pacific Islanders in the same age group has increased by 22.7% per year from 2017 to 2020 (95% CI: 6.0, 42.1).

Analysis by region showed worsening trends in the Northeast across all racial/ethnic subgroups, except among Non-Hispanic Blacks, whose trends remained stable from 2018 to 2020 (APC = 16.4; 95% CI: −0.8, 36.5). In the West, recent trends were stable among Hispanics since 1999, while increasing among all other racial/ethnic subgroups. The latest mortality trends in the Midwest and South increased among all racial/ethnic subgroups (Table 2).

## 4. Discussion

The results of this study reveal disparities in the burden of alcohol-related deaths based on race and ethnicity in the United States between 1999 and 2020. Notably, American Indians/Alaska Natives had the highest death burden, a trend consistent across sub-categories of age, sex, census region, and cause. This mirrors previous research, which has also reported disproportionate alcohol-related deaths by race and ethnicity, with American Indians/Alaska Natives suffering the highest burden [3,21,22,26]. This study, however, highlights a new emerging pattern: recent rates have relatively leveled among American Indians/Alaska Natives while increasing significantly among Non-Hispanic Whites, Non-Hispanic Blacks, Asians/Pacific Islanders, and Hispanics.

An assessment of alcohol and opioid-related deaths by race and ethnicity in Washington state between 2011 and 2017 found racial and ethnic differences, with American Indians/Alaska Natives and Hispanics having disproportionately higher rates than other racial and ethnic subgroups [22]. According to the CDC, American Indians/Alaska Natives also had the highest rate of opioid overdose deaths in 2019 and 2020 compared to all other racial/ethnic groups. Similarly, a study of racial and ethnic differences in smoking, drinking, and illicit drug use among 17,000 high school adolescents in the US between 1976 and 1989 found that American Indians/Alaska Natives had a higher burden of substance use compared to all other subgroups [26]. Keyes et al. [21] also reviewed evidence from 19 peer-reviewed publications and found that alcohol-related injuries are differentially distributed by race and ethnicity, with American Indians/Alaska Natives having the highest burden, particularly in terms of alcohol-related motor vehicle fatalities, suicides, and falls.

The findings of this study also indicate that the recent trend of alcohol-related mortality is generally deteriorating among all racial and ethnic sub-groups, except for American Indians/Alaska Natives, whose rates have remained constant. The reasons for this difference are not yet known. However, it is possible that efforts aimed at addressing this issue, such as the Native American Connections’ [NAC] Alcohol and Substance Abuse Prevention Program [27], the Native Voices implemented by the National Institute on Alcohol Abuse and Alcoholism [12], and the Alcohol and Substance Abuse Program implemented by the Indian Health Service [28], may have had a positive impact in recent years to at least counteract an increase in mortality rates, as seen with other racial/ethnic subgroups. While further research is required to determine the impact of these programs, there is an urgent need to go beyond stabilizing the trends and instead aim at reducing the overall alcohol-related mortality in American Indians/Alaska Natives, particularly by increasing adequate access to comprehensive healthcare services.

The disproportionate burden of alcohol-related deaths among American Indians/Alaska Natives may be due to social and economic factors that increase their vulnerability to poor health outcomes, such as poverty, unemployment, lower educational attainment, and lack of access to healthcare [3]. Additionally, American Indians/Alaska Natives have experienced historical colonization and cultural oppression, which has led to significant psychological and social trauma and, in turn, higher rates of substance abuse and poor health outcomes [29,30,31]. Mental health disorders, including addictions, can result from adverse social circumstances created by poverty and marginalization. There is accumulating evidence that environmental factors such as stress due to adversity and hardship can cause epigenetic changes, changes in gene expression, and changes in neural circuitry function that can trigger the development of mental health disorders, including addiction [32,33]. Cultural and social factors, such as the role of alcohol in traditional celebrations and ceremonies, may also play a role [34,35]. To effectively address this problem, research must identify the drivers of alcohol-related deaths among American Indians/Alaska Natives and inform culturally competent public health interventions.

Substance-use prevention programs can be an effective tool in reducing the disproportionate burden of alcohol-related deaths among American Indians/Alaska Natives, as well as other minority groups [36,37,38]. School-based prevention programs that provide youth with knowledge and skills to resist peer pressure and make healthy choices [39,40], as well as family-based programs that promote positive parenting practices and communication, have been shown to reduce substance use among youth [41]. Culturally grounded prevention programs that consider the unique cultural experiences and values of the targeted population may be particularly effective in addressing the underlying drivers of alcohol-related mortality among American Indians/Alaska Natives [42,43]. Therefore, future research should focus on the development and implementation of culturally tailored substance-use prevention programs for this population to reduce the disparities in alcohol-related mortality rates.

While our study focused on alcohol-related deaths in the US using underlying cause of death files, it is important to consider the impact of Fetal Alcohol Spectrum Disorder (FASD) in American Indians. Though not captured in our study, FASD is a significant public health problem for American Indians, with high rates and low life expectancy in affected individuals [44,45]. Future studies should explore the relationship between FASD and alcohol-related deaths in American Indians. Addressing underlying issues, including FASD prevention and treatment programs, can work towards reducing alcohol-related deaths and improving overall public health.

The present study has certain limitations that need to be considered. First, relying solely on alcohol-related deaths as the primary health outcome may overlook potential intervention opportunities at earlier stages of the health spectrum. Nonetheless, mortality data were utilized in this study due to the abundance of research on alcohol use, hospitalization, and care, as well as the pressing need for more information on racial/ethnic health disparities in alcohol mortality [17,18,19,20]. Second, the data used in this study were sourced from death certificate records of US residents and may contain inaccuracies in documenting the race/ethnicity of the deceased [46,47]. Third, the analysis presented in this study did not utilize place-based variables to examine spatiotemporal trends in alcohol-related mortality, which may limit the full understanding of geographic patterns and changes in alcohol-related mortality rates over time. While our study provides valuable insights into the racial and ethnic differences in alcohol-related mortality rates in the US, further research is necessary to explore these spatiotemporal trends and to better comprehend the underlying factors that contribute to them. Fourth, while our study found significant racial disparities in alcohol-related mortality rates among both males and females, further research is needed to examine sex differences in depth across different sociodemographic strata. This could help identify specific social and cultural factors that contribute to health disparities and inform targeted interventions to address them. Finally, data for this study were obtained from the CDC WONDER database, which only covers deaths from 1999 to 2020 [23]. Future studies should re-examine this analysis as new data cycles are released and consider incorporating additional health outcomes and variables to enhance the accuracy and comprehensiveness of the findings.

Despite the challenges associated with this study, its contribution lies in its utilization of national data to highlight the disparities in alcohol-related mortality rates based on race and ethnicity in the US. By shedding light on these inequities, the study underscores the significance of public health measures in addressing social vulnerability and improving health outcomes for all communities, regardless of race and ethnicity. These findings serve as a noteworthy addition to the existing body of literature on social vulnerability and emphasize the need for further action to promote health equity.

## 5. Conclusions

This study highlights the disparities in alcohol-related deaths based on race and ethnicity in the United States, with American Indians/Alaska Natives experiencing the highest burden. The results emphasize the need for targeted and region-specific interventions to address racial and ethnic disparities in alcohol-related deaths, as trends are not uniform across different age and regional sub-groups. Age-specific strategies are also required to effectively tackle the issue, as different age groups may have unique risk factors and protective factors related to alcohol use and abuse. Although the latest trend in alcohol-related mortality has generally worsened among all racial and ethnic groups, the rates for American Indians/Alaska Natives have remained constant in recent years. Further research is necessary to understand these disparities’ underlying causes and develop culturally sensitive interventions that address alcohol-related health outcomes equitably for all populations.

## Figures and Tables

**Table 1:** Racial/Ethnic Disparities in Alcohol Mortality Rates in the United States by Sex, Age, Region, and Cause, 1999–2020.

Variable ^a^	AAMR (95% CI) ^b^	Disparity Rate Ratio (95% CI) ^c^
Overall		
Non-Hispanic Black	7.1 (7.1–7.2)	0.68 (0.67, 0.68)
Asian/Pacific Islander	2.0 (1.9–2.0)	0.20 (0.19, 0.20)
American Indian/Alaska Native	38.7 (38.1–39.2)	3.62 (3.57, 3.67)
Hispanic	9.7 (9.6–9.8)	0.72 (0.72, 0.73)
Male		
Non-Hispanic Black	11.6 (11.5–11.8)	0.70 (0.69, 0.71)
Asian/Pacific Islander	3.5 (3.4–3.6)	0.22 (0.22, 0.23)
American Indian/Alaska Native	50.9 (50.0–51.8)	3.15 (3.09, 3.21)
Hispanic	16.9 (16.7–17.0)	0.80 (0.80, 0.81)
Female		
Non-Hispanic Black	3.6 (3.5–3.6)	0.65 (0.64, 0.66)
Asian/Pacific Islander	0.7 (0.7–0.7)	0.13 (0.13, 0.14)
American Indian/Alaska Native	27.6 (27.0–28.2)	4.84 (4.73, 4.96)
Hispanic	3.2 (3.1–3.3)	0.46 (0.45, 0.47)
Age (years)		
15–24		
Non-Hispanic Black	0.2 (0.2–0.2)	0.69 (0.60, 0.78)
Asian/Pacific Islander	0.1 (0.1–0.2)	0.42 (0.32, 0.54)
American Indian/Alaska Native	3.6 (3.2–3.9)	12.15 (10.80, 13.67)
Hispanic	0.5 (0.5–0.5)	1.70 (1.56, 1.84)
25–44		
Non-Hispanic Black	4.1 (4.0–4.1)	0.65 (0.64, 0.67)
Asian/Pacific Islander	1.7 (1.6–1.7)	0.27 (0.26, 0.28)
American Indian/Alaska Native	47.4 (46.2–48.5)	7.80 (7.61, 7.99)
Hispanic	6.1 (6.0–6.2)	0.96 (0.95, 0.98)
45–64		
Non-Hispanic Black	18.9 (18.7–19.1)	0.86 (0.85, 0.87)
Asian/Pacific Islander	4.4 (4.3–4.6)	0.20 (0.19, 0.21)
American Indian/Alaska Native	84.5 (82.9–86.1)	3.78 (3.71, 3.85)
Hispanic	23.8 (23.5–24.0)	1.06 (1.05, 1.07)
≥65		
Non-Hispanic Black	13.5 (13.3–13.8)	1.06 (1.04, 1.08)
Asian/Pacific Islander	3.7 (3.5–3.9)	0.28 (0.27, 0.30)
American Indian/Alaska Native	41.6 (39.8–43.4)	3.33 (3.19, 3.47)
Hispanic	20.2 (19.9–20.6)	1.56 (1.53, 1.59)
Census Region		
Northeast		
Non-Hispanic Black	6.5 (6.3–6.6)	0.84 (0.83, 0.86)
Asian/Pacific Islander	1.7 (1.6–1.8)	0.23 (0.22, 0.24)
American Indian/Alaska Native	8.5 (7.5–9.5)	1.14 (1.02, 1.28)
Midwest		
Non-Hispanic Black	8.0 (7.8–8.1)	0.85 (0.83, 0.87)
Asian/Pacific Islander	1.5 (1.3–1.6)	0.15 (0.14, 0.16)
American Indian/Alaska Native	39.5 (38.1–40.8)	4.13 (4.00, 4.27)
Hispanic	7.4 (7.2–7.6)	0.58 (0.56, 0.59)
South		
Non-Hispanic Black	6.6 (6.5–6.6)	0.65 (0.64, 0.66)
Asian/Pacific Islander	1.4 (1.3–1.5)	0.14 (0.13, 0.15)
American Indian/Alaska Native	13.7 (13.2–14.3)	1.42 (1.36, 1.48)
Hispanic	6.2 (6.1–6.3)	0.51 (0.50, 0.51)
West		
Non-Hispanic Black	10.6 (10.3–10.8)	0.65 (0.64, 0.67)
Asian/Pacific Islander	2.4 (2.3–2.4)	0.17 (0.16, 0.17)
American Indian/Alaska Native	62.0 (60.9–63.0)	3.73 (3.67, 3.80)
Hispanic	14.5 (14.3–14.6)	0.68 (0.67, 0.69)
Cause		
Alcohol Poisoning (Overdose)		
Non-Hispanic Black	0.4 (0.4–0.4)	0.71 (0.68, 0.74)
Asian/Pacific Islander	0.2 (0.1–0.2)	0.29 (0.26, 0.31)
American Indian/Alaska Native	4.2 (4.0–4.3)	7.51 (7.19, 7.84)
Hispanic	0.6 (0.6–0.6)	0.97 (0.94, 1.00)
All Other Causes		
Non-Hispanic Black	6.8 (6.7–6.8)	0.68 (0.67, 0.68)
Asian/Pacific Islander	1.8 (1.8–1.9)	0.19 (0.19, 0.20)
American Indian/Alaska Native	34.5 (34.0–35.0)	3.40 (3.35, 3.45)
Hispanic	9.1 (9.1–9.2)	0.71 (0.71, 0.72)

^a^ Hispanics could be of any race; all other categories are non-Hispanic. ^b^ Age adjusted Mortality Rate. ^c^ The referent is Non-Hispanic White. The 95% CI was computed using Taylor Series.

**Table 2:** Annual Percentage Changes (APC) and Average Annual Percentage Changes (AAPC) in Alcohol-Induced Mortality in the US, United States, 1999–2020.

Variable	Trend Segment	Segment Endpoints		APC (95% CI)	AAPC
		Lower	Upper		
Overall	1	1999	2007	0.0 (−0.6, −0.6)	2.9 (2.3, 3.4) *
	2	2007	2018	3.0 (2.6, 3.5) *	
	3	2018	2020	14.1 (8.2, 20.3) *	
Race/Ethnicity					
Non-Hispanic White	1	1999	2007	1.3 (0.8, 1.8) *	3.7 (3.2, 4.2) *
	2	2007	2018	3.7 (3.3, 4.1) *	
	3	2018	2020	14.3 (9.1, 19.9) *	
Non-Hispanic Black	1	1999	2007	−6.2 (−7.0, −5.3) *	−0.3 (−1.2, 0.6)
	2	2007	2018	1.2 (0.5, 1.9) *	
	3	2018	2020	17.0 (7.3, 27.5) *	
Asian/Pacific Islander	1	1999	2015	1.1 (0.1, 2.0) *	3.0 (1.6, 4.4) *
	2	2015	2020	9.5 (3.6, 15.6) *	
American Indian/Alaska Native	1	1999	2018	3.2 (2.7, 3.7) *	4.5 (2.9, 6.1) *
	2	2018	2020	17.9 (−0.3, 39.3)	
Hispanic	1	1999	2004	−3.8 (−6.0, −1.5) *	0.7 (−0.4, 1.8)
	2	2004	2018	0.7 (0.2, 1.3) *	
	3	2018	2020	12.6 (1.3, 25.1) *	
Sex & Race/Ethnicity					
Male					
Non-Hispanic White	1	1999	2009	1.1 (0.7, 1.5) *	3.2 (2.6, 3.8) *
	2	2009	2018	3.4 (2.8, 4.0) *	
	3	2018	2020	13.3 (7.3, 19.5) *	
Non-Hispanic Black	1	1999	2008	−5.7 (−6.7, −4.7) *	−0.6 (−1.8, 0.5)
	2	2008	2018	1.0 (−0.0, 2.1)	
	3	2018	2020	15.8 (3.3, 29.7) *	
Asian/Pacific Islander	1	1999	2016	1.2 (0.3, 2.1) *	2.9 (1.3, 4.5) *
	2	2016	2020	10.4 (2.0, 19.5) *	
American Indian/Alaska Native	1	1999	2006	−0.0 (−2.8, 2.9)	2.8 (1.7, 3.9) *
	2	2006	2020	4.2 (3.2, 5.3) *	
Hispanic	1	1999	2012	−1.5 (−2.2, −0.8) *	0.2 (−0.5, 0.9)
	2	2012	2020	3.1 (1.6, 4.7) *	
Female					
Non-Hispanic White	1	1999	2008	2.6 (2.1, 3.2) *	4.9 (4.3, 5.6) *
	2	2008	2018	5.1 (4.5, 5.7) *	
	3	2018	2020	15.0 (8.1, 22.4) *	
Non-Hispanic Black	1	1999	2007	−5.8 (−7.4, −4.2) *	0.7 (−0.9, 2.3)
	2	2007	2018	2.8 (1.6, 4.1) *	
	3	2018	2020	17.0 (0.3, 36.4) *	
Asian/Pacific Islander	1	1999	2003	−10.6 (−22.5, 3.2)	2.2 (−0.6, 5.1)
	2	2003	2020	5.5 (3.8, 7.2) *	
American Indian/Alaska Native	1	1999	2018	3.9 (3.5, 4.4) *	5.6 (4.2, 7.0) *
	2	2018	2020	22.8 (5.9, 42.4) *	
Hispanic	1	1999	2005	−1.7 (−5.2, 1.8)	1.5 (0.4, 2.7) *
	2	2005	2020	2.9 (2.0, 3.8) *	
Age (Years) & Race/Ethnicity					
15–24					
Non-Hispanic White	1	1999	2007	11.0 (7.2, 15.0) *	4.1 (0.8, 7.6) *
	2	2007	2018	−4.0 (−6.3, −1.6) *	
	3	2018	2020	26.0 (−8.6, 73.6)	
Hispanic	1	1999	2010	3.1 (1.0, 5.2) *	0.8 (−3.3, 5.2)
	2	2010	2013	−15.7 (−37.6, 13.8)	
	3	2013	2020	5.2 (1.1, 9.5) *	
25–44					
Non-Hispanic White	1	1999	2010	0.5 (−0.1, 1.0)	4.3 (3.4, 5.2) *
	2	2010	2018	5.1 (3.9, 6.3) *	
	3	2018	2020	24.4 (14.0, 35.7) *	
Non-Hispanic Black	1	1999	2009	−7.6 (−8.9, −6.2) *	0.6 (−1.4, 2.5)
	2	2009	2018	4.4 (2.3, 6.6) *	
	3	2018	2020	29.4 (7.0, 56.5) *	
Asian/Pacific Islander	1	1999	2011	0.7 (−1.0, 2.5)	5.0 (2.2, 7.9) *
	2	2011	2017	5.5 (−1.1, 12.7)	
	3	2017	2020	22.7 (6.0, 42.1) *	
American Indian/Alaska Native	1	1999	2003	−1.5 (−6.1, 3.3)	5.2 (3.5, 6.9) *
	2	2003	2018	4.7 (3.9, 5.4) *	
	3	2018	2020	24.3 (6.9, 44.6) *	
Hispanic	1	1999	2012	−1.8 (−2.5, −1.1) *	2.5 (1.0, 4.1) *
	2	2012	2018	5.3 (2.1, 8.6) *	
	3	2018	2020	25.4 (9.3, 43.8) *	
45–64					
Non-Hispanic White	1	1999	2018	3.3 (3.1, 3.4) *	4.1 (3.5, 4.6) *
	2	2018	2020	11.9 (5.5, 18.7) *	
Non-Hispanic Black	1	1999	2007	−5.7 (−6.4, −4.9) *	−0.6 (−1.3, 0.2)
	2	2007	2018	0.7 (0.1, 1.2) *	
	3	2018	2020	14.7 (6.9, 23.1 *)	
Asian/Pacific Islander	1	1999	2020	2.7 (2.0, 3.5) *	2.7 (2.0, 3.5) *
American Indian/Alaska Native	1	1999	2006	0.9 (−1.3, 3.2)	3.5 (2.6, 4.4) *
	2	2006	2020	4.8 (4.0, 5.6) *	
Hispanic	1	1999	2004	−3.1 (−4.6, −1.7) *	0.4 (−0.3, 1.1)
	2	2004	2018	0.7 (0.3, 1.0) *	
	3	2018	2020	7.4 (0.6, 14.7) *	
≥65					
Non-Hispanic White	1	1999	2011	0.1 (−0.4, 0.7)	2.6 (2.2, 3.0) *
	2	2011	2020	6.0 (5.2, 6.9) *	
Non-Hispanic Black	1	1999	2003	−7.6 (−12.0, −3.1) *	−0.6 (−1.8, 0.6)
	2	2003	2011	−1.8 (−3.7, 0.3)	
	3	2011	2020	3.7 (2.3, 5.2) *	
Census Region & Race/Ethnicity					
Northeast					
Non-Hispanic White	1	1999	2005	0.5 (−0.6, 1.7)	3.7 (2.9, 4.4) *
	2	2005	2018	3.6 (3.2, 4.1) *	
	3	2018	2020	13.9 (6.3, 22.0) *	
Non-Hispanic Black	1	1999	2005	−7.6 (−10.0, −5.0) *	−0.2 (−1.9, 1.4)
	2	2005	2018	0.9 (−0.0, 1.9)	
	3	2018	2020	16.4 (−0.8, 36.5)	
Asian/Pacific Islander	1	1999	2020	1.8 (0.6, 3.0) *	1.8 (0.6, 3.0) *
Hispanic	1	1999	2007	−5.4 (−7.8, −3.0) *	−0.5 (−1.7, 0.6)
	2	2007	2020	2.6 (1.3, 3.8) *	
Midwest					
Non-Hispanic White	1	1999	2007	1.4 (0.5, 2.4) *	4.5 (3.6, 5.4) *
	2	2007	2018	4.6 (3.9, 5.3) *	
	3	2018	2020	17.5 (8.0, 27.9) *	
Non-Hispanic Black	1	1999	2007	−6.2 (−7.9, −4.4) *	0.9 (−0.8, 2.7)
	2	2007	2018	3.6 (2.2, 5.0) *	
	3	2018	2020	17.2 (−1.1, 38.9)	
American Indian/Alaska Native	1	1999	2001	−8.3 (−24.0, 10.5)	3.7 (1.5, 5.9) *
	2	2001	2017	2.4 (1.6, 3.2) *	
	3	2017	2020	20.2 (9.5, 32.0) *	
Hispanic	1	1999	2003	−11.6 (−18.7, −3.9) *	1.2 (−0.4, 2.9)
	2	2003	2020	4.5 (3.5, 5.5) *	
South					
Non-Hispanic White	1	1999	2011	1.2 (0.5, 1.8) *	3.4 (2.8, 3.9) *
	2	2011	2020	6.4 (5.4, 7.5) *	
Non-Hispanic Black	1	1999	2009	−6.0 (−6.8, −5.2) *	−0.8 (−1.9, 0.3)
	2	2009	2018	1.4 (0.2, 2.7) *	
	3	2018	2020	17.7 (5.4, 31.5) *	
American Indian/Alaska Native	1	1999	2020	3.2 (2.3, 4.2) *	3.2 (2.3, 4.2) *
Hispanic	1	1999	2011	−2.0 (−3.1, −0.9) *	1.0 (0.1, 1.9) *
	2	2011	2020	5.1 (3.3, 7.0) *	
West					
Non-Hispanic White	1	1999	2018	2.2 (2.0, 2.4) *	3.0 (2.4, 3.7) *
	2	2018	2020	11.6 (4.5, 19.2) *	
Non-Hispanic Black	1	1999	2010	−2.3 (−3.4, −1.1) *	0.1 (−0.7, 1.0)
	2	2010	2020	2.8 (1.4, 4.2) *	
Asian American/Pacific Islander	1	1999	2020	2.3 (1.5, 3.2) *	2.3 (1.5, 3.2) *
American Indian/Alaska Native	1	1999	2020	4.0 (3.4, 4.5) *	4.0 (3.4, 4.5) *
Hispanic	1	1999	2018	−0.1 (−0.5, 0.3)	0.9 (−0.3, 2.1)
	2	2018	2020	10.7 (−2.8, 26.1)	

* *p*-value < 0.05; 95% confidence interval does not include 0.

## Data Availability

The data presented in this study are openly available at https://wonder.cdc.gov/ (accessed on 19 April 2023).
